# Supplementary material for: The interactome of intact mitochondria by cross-linking mass spectrometry provides evidence for coexisting respiratory supercomplexes
Source: Mol Cell Proteomics. 2017 Dec 8;17(2):216–32. doi: 10.1074/mcp.RA117.000470 (PMC5795388; doi:10.1074/mcp.RA117.000470)
Supplement: Supplemental Data [file supp_RA117.000470_133922_0_supp_23978_fzffwf.zip › spectra_annotation/mito_spectra_annotation/124-1-10-1-5-1.pdf]

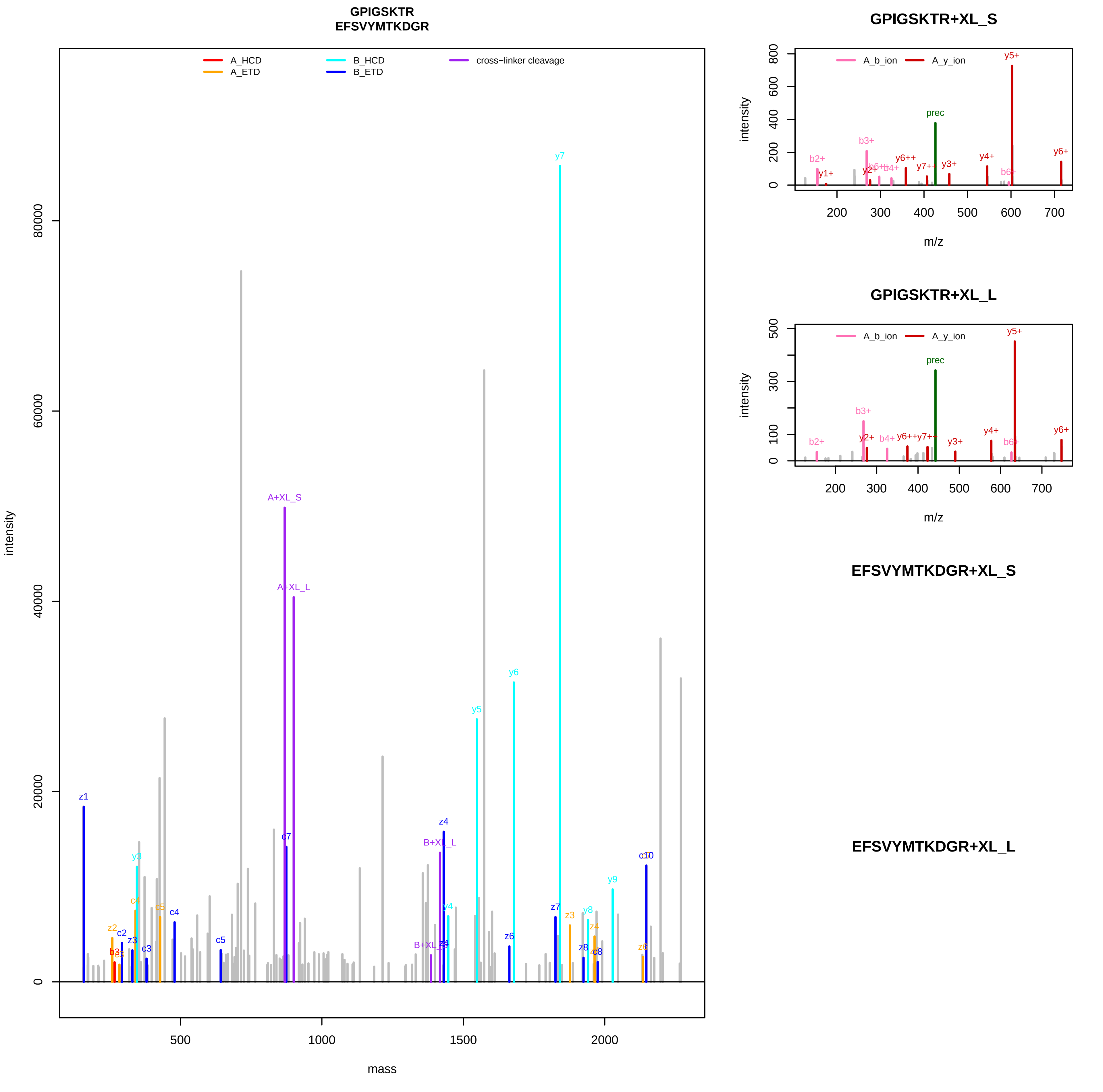
GPIGSKTR
EFSVYMTKDGR
A_HCD
A_ETD
B_HCD
B_ETD
cross−linker cleavage
intensity
mass
A+XL_S
A+XL_L
B+XL_L
y7
y6
y5
y9
y8
y4
y3
z1
z2
z3
z4
z6
z7
z8
c2
c3
c4
c5
c7
c8
c10
GPIGSKTR+XL_S
GPIGSKTR+XL_L
EFSVYMTKDGR+XL_S
EFSVYMTKDGR+XL_L
A_b_ion
A_y_ion
prec
y5+
y6+
y4+
y3+
y6++
y7++
y2+
y1+
b2+
b3+
b4+
b6+
m/z
